# Asclepion of Epidaurus: the application of a historical perspective in medical education

**DOI:** 10.1186/s13010-022-00120-6

**Published:** 2022-04-20

**Authors:** Charalabos Papageorgiou, Gerasimos Konstantinou, Vassilis Lambrinoudakis, Christos Papageorgiou, Konstantina G. Yiannopoulou

**Affiliations:** 1 1St Department of Psychiatry, School of Medicine, National and Kapodistrian University of Athens, Athens, Greece; 2 University Mental Health Research Institute (UMHRI), Athens, Greece; 3 Department of Classical Archaeology, National and Kapodistrian University of Athens, Athens, Greece; 4 Department of Clinical Therapeutics, Medical School, National and Kapodistrian University of Athens, Athens, Greece; 5 Department of Neurology, Henry Dunant Hospital Center, Athens, Greece

**Keywords:** Asclepion, Healing environment, Sacred space, Medical education, Medical ethics

## Abstract

**Background:**

The Asclepion of Epidaurus is one of the first healing environments in the world. Descendants of Asclepius, specifically medical students, have been singularly deprived of any information concerning this legacy. This article illuminates the role of Asclepion of Epidaurus and examines the view of medical students upon the subject and the possible benefits of this knowledge in their medical education.

**Methods:**

The participants were 105 senior-year students from the Athens Medical School, who attended a multi-media assisted lecture related to the structure and the role of the Asclepion of Epidaurus. Afterwards, they answered anonymously a questionnaire of 12 pairs of opposite adjectives in order to describe their view regarding the meaning of Asclepion. The method used in the evaluation of their answers was that of semantic differential.

**Results:**

The attitude of the students towards the meaning of Asclepion was positive, showing interest and excitement about a powerful, though unfamiliar piece of knowledge.

**Conclusion:**

Today’s novice doctors have welcomed the concept of Asclepion as essential knowledge for the service they will be called to fulfill. The potential benefits of the Asclepian ideals in medical education and ethos are thoroughly discussed.

## Background

Medical education nowadays calls for a reform due to the increasing ageing population and the rapidly growing number of patients with long term diseases and multiple comorbidities. Specialization and subspecialization which dominate in current models of medical training do not adequately prepare physicians to respond to patient and community needs anymore. Generalism *and *generalist* training* have been proposed as a potent solution to reforming the medical education in order to respond *to future population health needs.* Generalism includes uninterrupted and interpretative care, principles of whole-person focus, and first contact care for a wide range of situations and symptoms. Specialists, who are necessary to provide condition-focused solutions, may also integrate some features of generalist practice, such as the person-centred decision making [1].

Furthermore, increased health care needs and cultural diversity in modern societies demand a more sophisticated medical education which can transform all medical students to well-rounded personalities with the potential to play any one of the six key roles of the ideal doctor: communicator, team member, leader, health care advocate, professional and medical expert [2].

### Potential contribution of the Asclepions in the modern medical education

Ancient Greek medicine, as it was applied in the sacred spaces of Asclepions, may teach us about performing these key roles of the ideal modern doctor and generalism as well.

The Asclepions were sacred worshipping grounds οf doctor and healing god Asclepius. In fact, they were the first healing spaces, or rather building complexes, where medical services were rendered, not only on Greek land but also throughout western civilization [3]. From ancient written accounts as well as excavated remains, it is known today that the sacred spaces named “Asclepions” were equipped with mythological, ideological and philosophical perceptions of the well-being of our ancestors, offered holistic health care to their patients. A person (patient and supplicant) was treated with solemn respect, as a complete entity with inseparable spiritual, mental, emotional, social, moral and natural characteristics. Illness was viewed as the outcome of complex, negative interactions of environmental, social, psychological, spiritual, emotional and natural factors, and health care seemed to aim at resolving these conflicts and restoring balance among the above-mentioned by having as supplementary aid medical intervention, either surgical or pharmaceutical [4].

Today it is known from proof of written sources and/or archaeological findings the existence of approximately 320 Asclepions buildings in ancient Greece. In the mainland of Epidaurus, an area with mild climate and plentiful, therapeutic water springs is found perhaps the most significant therapeutic center in all of Greece and Rome: the Asclepion of Epidaurus. Although it was the main sanctuary of a small, seaside town of the Argolis region, its reputation and recognition quickly went beyond its geographical boundaries and was considered by the Greeks as the place where medicine was born. More than 100 therapeutic spring centers in the entire east Mediterranean are considered its foundings. Today these monuments are not only recognized as worldwide wonders of ancient Greek art and architecture, but also as exceptional testaments to the practice of medicine in antiquity. These monuments reflect the evolution of medical practice, starting from the time curing illness was dependent wholly on a god to its transformation into a science which involved the systematic recording of medical cases and the gradual gathering of knowledge and experience [5].

Nowadays, the Asclepion is considered to be the primary form of holistic medical design and is studied in depth by Greek and international centers as a sacred prototype of incredible significance and is used as a guidance for new medical design proposals [4, 5].

However, the question arises of whether descendants of Asclepius, more specifically, medical students and new doctors, recognize the significance of this legacy and what their view is upon the subject.

In answer to the above, graduates of the Medical School of Athens were called to complete a questionnaire. The method used in the evaluation of their answers was that of semantic differential [6], a very simple method developed by Charles Osgood, 67 years ago [7]. The semantic differential is probably the most successful empirical method invented for studying the nature of connotative meaning of any concept [8].

The semantic differential scale is considered to be a special version of a cumulative grading scale, which is used for measuring the importance of meanings. The purpose for creating this scale was for a quantitative description of different ways (subjective) a person interprets a specific concept. The main assumption of this method is that the interpretation of a concept is based on the attributes each person assigns to it through his personal experiences and not through what is socially acceptable or objective [7].

Establishing the semantic differential method is procedurally plain: Concepts (e.g. settings, actions, behaviors, techniques, objects) are presented to participants who are asked to rate them on perhaps as many as 10—50 scales [9]. Each scale is typically a seven point scale based on opposing adjectives (e.g. good-bad, fair-unfair, etc.), with the central point being neutral. The participants assess the concept under study by using these adjectives. Usually a number of 10–20 pairs of adjectives are considered satisfactory to examine all aspects of the phenomenon. A greater number would lead to participant fatigue [6].

Osgood studied fundamental dimensions that could lead to a differentiation of perception that participants have of a concept. He submitted for analysis the answers of one group of participants with 50 bipolar pairs of adjectives (e.g. fair-unfair, good-bad, strong–weak, fast-slow). Factor analysis or principal components analysis was then used to determine the concealed dimensions or factors underlying these ratings [10].

The results of this study revealed three aspects which were confirmed after subsequent research of different groups and societies [11]. The factors that resulted from this study are the following:

- **Activity**. It indicates the degree to which a concept is described by motion and action and captures the extent this concept is ‘active’ or ‘passive’. This is represented by adjectives such as active–passive, hot–cold, fast-slow.
- **Evaluation**. This aspect indicates the degree to which a concept is favorable to an individual. It almost always corresponds to whether the concept is ‘good’ or ‘bad’. It is represented by pairs of adjectives such as valuable-useless, sacred-sacrilegious, good-bad, clean-dirty, pleasant-unpleasant.
- **Potency**. This aspect captures the extent the concept is ‘strong’ or ‘weak’. It is represented by adjectives such as strong–weak, heavy-light, big-small.

The technique of semantic differential is a useful means to create scales for research purposes and multiple applications. The scale is used broadly for the measurement of the attitude one has towards persons, social groups, institutions, social phenomena, etc. as well as the detection of differences in the way individuals perceive particular concepts. The way in which a participant evaluates a concept (e.g. valuable-useless, strong–weak, good-bad etc.) expresses a more general attitude towards it [6, 7].

Although the semantic differential method is over sixty years old, it has been found to be vigorous across cultures [11] and domains [12], probably because the three dimensions of the semantic differential represent a summary of the universal human perception, specifically the average sense of order, benefit and power associated with a particular concept.

## Methods

The participants were 105 senior-year students from the Athens University Medical School (46 women and 47 men). Participation was voluntary.

Within the context of the Psychiatry course, an introduction was given through multi-media relating to the structure and the role of the Asclepion of Epidaurus. All of the students were unaware of the existence of an Asclepion in the ancient region of Epidaurus and most of them had a vague idea of the role of the Asclepions as healing spaces and their contribution in the evolution of medical practice.

The scientific-rational and mystical -divine healing systems that coexisted in a dynamic balance in these healing spaces were analyzed in the introduction. The students became aware of the healing method of Asclepions where besides relieving, a broader scope was required: one in which questions of meaning, purpose, destiny and mortality could be more fully explored and individuals were strongly bound by frameworks of nature and community. It was also highlighted that illness was considered within the context of spirituality instead of physical failing. Besides some attention to the bodily frame, the power of the mind was unleashed through images, stories, sensations, and "radical rituals" designed to break down old assumptions.

After the introduction, the following questionnaire was distributed and requested that the participants fill it out anonymously:

**Table:** 

**PLEASANT**	+ 3	+ 2	+ 1	0	-1	-2	-3	**UNPLEASANT**
BIG	+ 3	+ 2	+ 1	0	-1	-2	-3	**SMALL**
FAST	+ 3	+ 2	+ 1	0	-1	-2	-3	**SLOW**
GOOD	+ 3	+ 2	+ 1	0	-1	-2	-3	**BAD**
STRONG	+ 3	+ 2	+ 1	0	-1	-2	-3	**WEAK**
ALIVE	+ 3	+ 2	+ 1	0	-1	-2	-3	**DEAD**
SWEET	+ 3	+ 2	+ 1	0	-1	-2	-3	**SOUR**
POSSIBLE	+ 3	+ 2	+ 1	0	-1	-2	-3	**IMPOSSIBLE**
QUIET	+ 3	+ 2	+ 1	0	-1	-2	-3	**NOISY**
USEFUL	+ 3	+ 2	+ 1	0	-1	-2	-3	**USELESS**
MEANINGFUL	+ 3	+ 2	+ 1	0	-1	-2	-3	**SENSELESS**
YOUNG	+ 3	+ 2	+ 1	0	-1	-2	-3	**OLD**

The questionnaire consisted of basic demographic information of the participants (age, sex) as well as 12 pairs of adjectives: pleasant-unpleasant, big-small, good-bad, fast-slow, possible-impossible, strong–weak, alive-dead, sweet–sour, noisy-quiet, useful-useless, meaningful-senseless, young-old. For selecting the most appropriate pairs of adjectives for our study, we used the semantic differential dictionary which was compiled during 2002/3 at Indiana University [13]. This dictionary consists of 1500 concepts grouped under four different headings: Behaviors (actions that a person can perform), Identities (different kinds of individual), Settings (places or times where interactions might take place) and Modifiers (emotions, traits, and statuses). We chose the final 12 pairs of adjectives among them under the heading of Settings. Each pair contained two poles of an attribute, among which were placed 7 values (-3, -2, –1, 0, + 1, + 2, + 3).

The subjects of this research were called to describe their view regarding the meaning of “Asclepion” under the scope of their subjective point of view and from the introduction that was conducted. Since they were totally unaware of the meaning and the healing role of the Asclepion of Epidaurus and the Asclepions in general, before the lecture, their knowledge and impressions were totally new and vivid and it was almost spontaneous for them to assign a number in the 7 point scale for each bipolar pair corresponding to their personal view about the characterization of Asclepions as a setting pleasant or unpleasant, big or small, good or bad, fast or slow, possible or impossible, strong or weak, alive or dead, sweet or sour, noisy or quiet, useful or useless, meaningful or senseless, young or old and to what extent. A score between -3 and -1 represents an attitude more proximal to the negative pole of the pair (for instance, for the pair unpleasant – pleasant, the participant percepts this setting as unpleasant), a score of 0 represents a neutral response (neither unpleasant or pleasant), and a score + 1 to + 3 represents an attitude more proximal to the positive pole (more pleasant than unpleasant).

After the questionnaires were collected, the data was subsequently recorded on an Excel sheet followed by a factor analysis with the purpose of defining the dimensions of meaning under examination and the individual properties. Finally, calculations were made on the overall score of each participant by adding the numerical answers to the adjectives that represent each dimension. The seven-number scale was modified as -3, -2, -1, 0, 1, 2, 3. The negative scoring concerned greater proximity to the negative pole of each pair.

## Results

**Fig. 1:**
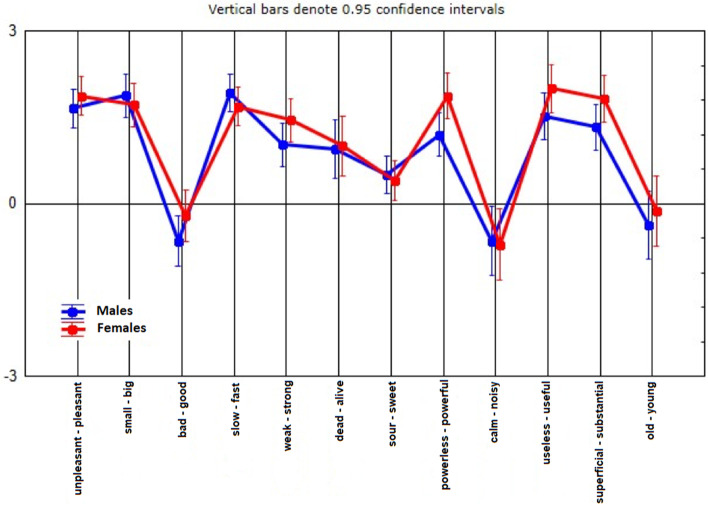
Depiction of the average value for each dimension by gender: Following the recording of the score, each participant assigned to every dimension, the numerical answers were added and the average value for each dimension by gender was calculated

Following the recording of the score each participant assigned to every dimension, the numerical answers were added and the mean value for each dimension by gender was calculated (Fig. 1). Overall there were no noticeable differences between the sexes (Table 1). Both women’s and men’s answers were directed towards the negative pole only in three cases of bipolar pairs. The specific bipolars were: slow-fast, old-young, noisy-quiet. In the remaining bipolar pairs the scores tended to lean towards the positive side for both genders (Fig. 1).

**Table 1:** No significant differences between the sexes were remarked by the Mann–Whitney U inferential statistics test

Mann–Whitney U test	Rank Sum men	Rank Sum women	U	*p*-value
Unpleasant—Pleasant	1804,500	1936,500	814,5000	0,346,354
Small—Big	2081,500	2013,500	953,5000	0,648,111
Bad—Good	1853,000	1888,000	818,0000	0,368,536
Slow – Fast	2211,000	1794,000	848,0000	0,248,690
Weak – Strong	1964,000	1952,000	883,0000	0,490,697
Dead—Alive	2122,000	1973,000	994,0000	0,897,166
Sour – Sweet	2232,500	1772,500	826,5000	0,183,499
Powerless—Powerfull	1866,500	2049,500	831,5000	0,258,021
Noisy – Quiet	2106,500	1809,500	906,5000	0,622,095
Useless—Useful	1922,000	2173,000	794,0000	0,081,024
Superficial—Substantial	1880,000	2036,000	799,0000	0,164,247
Old—Young	2056,500	1948,500	928,5000	0,633,593

Principal component analysis with varimax rotation performed on the questionnaire data, revealed the presence of three factors. These factors explained the 51,24% of total variance. The first of the three factors (Activity) accounted for 20.89% of the total variance, the second (Evaluation) for 16.40% while the third (Potency) for 13.94%.

The first factor (Activity) had high loadings on the four bipolar pairs: fast-slow, powerful-powerless, useful-useless, substantial-superficial. These results show that the students perceived the healing environment of Asclepion as a dynamic process with rapidity, vitality, strength, positive influence and perpetual importance.

With respect to the second factor, high loadings were noted on the six pairs of bipolar adjectives: pleasant-unpleasant, good-bad, alive-dead, sweet–sour, noisy-quiet, young-old. The students’ answers described a rather modern meaning of the Asclepions, capable of creating pleasure, positivity, hope, vibration and even freshness.

Finally, the third factor had high loadings on the four bipolar pairs: pleasant-unpleasant, big-small, strong–weak, powerful-powerless (Table 2). This group of answers seems to depict an innovative educational and medical environment and it is a real surprise that in fact depicts an ancient healing world.

**Table 2:**
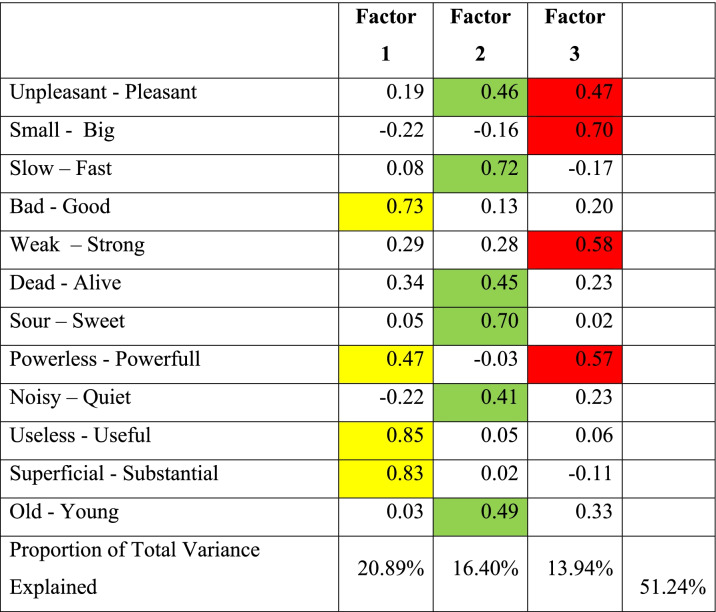
From the factor analysis that was conducted from the questionnaires, three factors emerged which accounted for 51.24% of the total deviation

Factor 1 (Activity) had high loadings in fast-slow, strong–weak, useful-useless, substantial-superficial and accounted for 20.89% of the total variance

Factor 2 (Evaluation) had high loadings in pleasant-unpleasant, good-bad, alive-dead, sweet–sour, noisy-calm, young-old and accounted for 16.40% of the total variance

Factor 3 (Potency) had high loadings in pleasant-unpleasant, big-small, strong–weak, powerful-powerless and accounted for 13.94% of the total variance

The highest percentages are highlighted

Overall, these results reveal that the majority of the students perceived the meaning and the role of Asclepion as an active (Activity factor), valuable (Evaluation factor) and powerful (Potency factor) entity. The most popular positive characterizations of Asclepion among the students, as they are depicted in Fig. 1 are: big, good, powerful, useful and substantial.

## Discussion

In the present study and in the context of a theoretical Psychiatry course given at the Medical School of Athens, graduating students studied the structure, role and organization of the Asclepion in Epidaurus, as mentioned before. Next, based on the introduction, their personal knowledge and subjective views of the Asclepion, the participants were called in to score in the form of a questionnaire the information they received, approaching the concept of “Asclepion” through the method of semantic differential.

From the analysis of the participants’ answers stemming from the questionnaire, it appears that for the descendants (novice doctors) of Asclepius’s work, the importance of the monument and its vital role is new information. Most likely, the concept of “Asclepion” might not be unprecedented. However, its connection to the archaeological site of Epidaurus, as well as the widespread impact it had for the times then, are what had been introduced for the first time. Today’s new doctors have welcomed the above as essential and useful knowledge. That effectively transferred knowledge has aroused their interest not only in the history and evolution of Asclepion, but also in the service, they will be called to fulfill.

Overall, the students showed a real interest and even excitement about this unfamiliar but attractive piece of knowledge. They also expressed their surprise with many questions and comments about the specific methods involved in this healing attitude, the results and the publicity of the sacred space of Asclepion of Epidaurus. During this survey, they had also the opportunity to confess that are no longer satisfied with the intensifying focus of scientific medicine as applied biology and technology and many demand the inclusion of spirituality in health education and care.

### Overview of healing environment and methods of Asclepion

The following piece of information helped them to complete their perception of the healing environment and methods of Asclepion and to connect it with the humanism and spirituality that the modern medical care demands.

About 2,500 years ago, the Greeks realised the necessity of a healing space in order to mend their society’s collective health and every citizen’s well-being since they had been at continual war for centuries. They selected Epidaurus as their building site for their healing sanctuary as they knew clearly what kind of residences and environments suited their needs. Indeed, the location of Epidaurus incorporates the two most well-established principles of current healthcare architecture: the inclusion of sunlight and that of natural scenery [5, 14, 15].

The pleasure every visitor derived from viewing the land of Epidaurus then extends to those experiencing its beautiful scenery today, and explains why the Asclepion was set up there. The mild climate along with the calming green foliage would have supplied the ill and cheerless pilgrims with recreation and tranquillity. Furthermore, the plentiful spring waters of the region were another chief asset in the healing expectation [5, 14, 15].

A well-organized list of active and passive rituals and healing activities was customized to the needs of every individual [16]. The various healing dimensions that were offered were:

- **The purification process:** “*Purity means to think nothing but holy thoughts*” [16] was the inscription with which the pilgrims/patients were encountered at the entrance of the sacred space. Basins for hygiene purposes and relaxation were the initial part of the purification process which would be lengthened by sacrifices and accompanying rituals.
- **Sacred healing rituals:** After purification, the directive priests created a state of deep auto—suggestion accompanied with religious exaltations in the patients-believers. The spiritual experience was further intensified by the healing aesthetic of sacred environments as well as the hymns chanted by the Paianists (specialised singers) [16].
- **Therapeutic procedures:** In separate(d) halls, the healing priests – ancient Greek physicians, also performed and recorded a detailed history and physical examination of the patient and afterwards, they recommended dietary guidelines, gave instructions for the use of many kinds of medicinal herbs per os or in the form of suppositories, ointments or eye drops and whenever it was necessary, they performed surgical operations [16, 17].
- **Physical exercise:** It took place in the gymnasium [5].
- **Communal bonding and encouragement:** This involved viewing athletic events in the stadium [5].
- **Communal catharsis:** It took place in the magnificent Epidaurus theatre with a seating capacity of 14,000, where ancient drama was presented [5].
- **Communal meals:** Formal banquets were included in the healing process for selected worshippers which took place in Banqueting Hall- a mesmerizing building that inspired pilgrims to trust each other and share empathy and hope in their common effort [18, 19].
- **Dream healing (“Enkoimesis”):** It was the final stage of the healing process with its own sacred space, which was named “Abaton” or “Enkoimeterion”. According to the priests, when the testing of the soul was complete, the believer was led to the sacred site to spend the night alone. Already overcome with religious faith and anticipation, the patient excitedly imagined the impending miracle of being cured. Moreover, concealed oil lamps which illuminated the premises, heightened even further the supernatural experience he was about to undergo. After the withdrawal of the priest, the patient was abandoned in the darkness to enter the phase of healing sleep. The following morning the patient would awake healthy [5, 14, 20].
- **Testimonials of gratitude:** After the cure, the worshipping of the god Asclepius in the monuments followed [14].

Contemporary psychologists and psychotherapists suggest that Asclepian purification and healing rituals when used shifted believers’ understanding of the self and the universe to the point where they could self-heal themselves and hence function in a healthy manner [20, 21].

The multitude of sacred spaces and ritual opportunities available at Epidaurus contributed to the ancient city’s reputation as a place for healing among traumatized pilgrims.

Overall, in ancient times the sanctuary of Asclepius was an organized medical healing center, which marked the transition from occult rituals for treatment to the use of actual medical science. That is, the therapy for any ailment would be executed based on diagnosis and appropriate administering of medicine or surgical intervention, but with divine guidance. It can be realized that medical practice in Asclepion of Epidaurus was focused simultaneously on a holistic and individual approach for every patient. At the same time, the issue of total harmony among the personal, social and natural environment was highlighted as a prioritized healing process within the boundaries of that sacred space [22].

The reputation of the Asclepion for the time then could be characterized as worldwide. With the Asclepion of Epidaurus being the center for patient care, reverence for Asclepius was spread not only in Greece but also throughout the Mediterranean world; and at the same time, with the creation of more than 100 subsidiary Asclepions, medical science was spread throughout Attica, in the Peloponnese, in western Greece, the Aegean Islands, Crete, from Asia Minor to the distant Cilicia, Rome and Cyrene in Africa. However, nowadays, the restoration and use of the ancient theater has led to the public’s mistaken perception of the archaeological grounds [3].

To our knowledge, there are no studies on the interest and views of doctors about the beginnings of medical practice in the Asclepions, especially about the Asclepion of Epidaurus. This site is not known as a great-sized monument of worship and medical practice as it had been, but as a great architectural and aesthetic, theatrical structure.

It is suggested, that the study of the healing environment of the Asclepion in Epidaurus can offer a plethora of benefits to novice doctors since they are eager to gain new ancient knowledge.

The results of our study reveal that the majority of the students perceived the Asclepion as an active setting (Activity factor). This remark can become a motivation for their educators to revive the most intriguing features of the Asclepions integrating them in modern medical education. The following suggestions can function as triggers for innovative educational methods based in the humanistic and holistic ideals of Asclepions:

First of all, medical students can appreciate the healing power of architecturally appealing and sun-filled surroundings that respect patients and the quality of their stay there. A specialized subject, such as medical architecture or architecture of healing environments could be proved beneficial for medical students as well as their future patients. It is true that nowadays modern medical centers’ design has shifted to a focus on patient, upgrading their surroundings aesthetically to provide a pleasant and relaxing experience for their patients and their families. However, the environment would not be defined as one of healing as those of the wise ancient Greeks had foreseen. Since their first impression of the Asclepion setting was that this space is active and powerful, the medical students’ curriculum could be pleasantly enriched by a brief subject about the main architectural features of sacred spaces and the healing powers of light and the views of natural environment [5, 23]. Hence, novice doctors can request the improvement of medical facilities in their working environment, where the model healing space of the Asclepion of Epidaurus would be followed. Ideally, organized visits to the Asclepion of Epidaurus could enlighten the natural impact of the architecture and the surroundings on their mentality. After that, they would probably seek the reproduction of the harmony, simplicity and grandeur of this environment to their everyday working space. Even more beneficial might be the educational workshops or small group congresses in this space. Deprived from any modern equipment, just talking and walking under the olive trees in small groups, accompanied by an experienced clinician who would provide them with facts and questions and totally concentrated in the conversation, it is more than probable that their appreciation for their role as therapists and for the active and continuous learning for their patients’ sake would become an invaluable lifetime’s lesson. To sum up, specialized subject, such as medical architecture or architecture of healing environments could be proved beneficial for medical students as well as their future patients.

Furthermore, the majority of the students, according to our study’s results perceived the Asclepion as a valuable setting (Evaluation factor). This aspect might be translated into their need for discovering deeper values in their profession, derived from the dawning of medicine in the healing spaces of the Asclepions.

For instance, modern medical educators inspired by priest – physicians of Asclepions can teach new doctors – through vivid presentations or documentaries to start thinking not only with guidelines, rules, management systems, and frameworks, as they have already been taught to do so, but also with phronesis. For Aristotle, phronesis, which means ‘practical wisdom’, is one of the most important human virtues. It commands our skills, thoughts, and courage, with the use of both knowledge and judgment, guiding us to the most efficient actions. Medical students and new doctors can emulate this kind of practical wisdom from the best practices of the ancient physicians, who worked in demanding and hostile environment, usually in times of warfare, without protocols or equipment, but always providing the best they could [24]. These priests – physicians had in mind that the real world always presents us with situations that are more complex than our experience and knowledge can predict and that patients have varying priorities and demand different therapeutic approaches to their being healed. And to achieve positive results, they worked with phronesis [25–27]. Modern educators, especially experienced clinicians in the emergencies or in remote areas without the basic technological facilities can effectively teach ‘phronesis’ in medical students.

The results of our study also indicated that medical students perceived the Asclepion as a powerful setting (Potency factor). Their estimation is probably in line with that of Socrates [28]:

The last words of the famous philosopher before his death, undoubtedly express what Asclepius’ values meant to the former personally.

“Crito, we owe a cock to Asclepius—Pay it and do not neglect it” [29].

Socrates had called his students for an offering to Asclepius. He was sentenced to death for impiety, that is, for not believing in the state’s gods but introducing new ones. Thus, the questions arises of why the philosopher’s last words were an expression of gratitude to a healing god.

The most probable explanation may be he believed in the reviving and humanistic power of Asclepian ideals, which was the only value that could keep his own spirit and ideas alive. Socrates summons us from the past to apply eternal Asclepian medical practices to self, patients and community. He reminds us of our personal duty to make our ethos a first priority, of our professional duty to alleviate suffering, and of our social duty to support the vulnerable, sick, and deprived [30].

Today, patients do not admire the focus of scientific medicine as applied technology on their bodies any more and many demand ethos, “phronesis” and the inclusion of spirituality in health care. Their request for meaningful answers to their eternal questions recurs: Why is this suffering happening only to me and now? Am I going to live and for how long? What is the meaning of my short life? What will be my legacy? Current medical education has to expand its scope and address the issue of suffering. The model of Asclepian healing spaces and methods enlightens this approach and can guide the modern medical education in this area.

The attitude of the students towards the new knowledge of Asclepion of Epidaurus was positive, showing a real interest and sometimes excitement about a useful and alive, though still unfamiliar piece of knowledge. We cannot ignore this attitude that allows us to see that our students are no longer satisfied with the intensifying focus of scientific medicine as applied biology and technology and many demand the inclusion of spirituality in health education and care.

We suggest that the modern curricula of medical students should be further potentiated by humanistic and social subjects, such as principles of philosophy, medical ethics and medical leadership. Thus, novice doctors might be empowered to treat not only diseases but also patients.

## Conclusions

We believe, as Socrates probably did, that it would be of great importance instead of focusing on our technocratic and selfish way of thinking to seek knowledge and points of perspectives of the Asclepions, especially that of Epidaurus, *which used the influence of environment and architecture and the principles of phronesis and ethos as healing powers.* The positive attitude of medical students to the meaning of Asclepions as healing spaces is a strong encouragement to further explore this source of medical experience and inspiration as an educational tool and environment. Future studies could evaluate the shift of view that this knowledge can induce in physicians’ way of thinking and practice of medical healing as well as the influence this shift can have on their patients’ satisfaction and overall health status.

## Data Availability

The datasets used and analyzed during the current study are available from the corresponding author on reasonable request.
